# Evaluation of upper extremity neurorehabilitation using technology: a European Delphi consensus study within the EU COST Action Network on Robotics for Neurorehabilitation

**DOI:** 10.1186/s12984-016-0192-z

**Published:** 2016-09-23

**Authors:** Ann-Marie Hughes, Sofia Barbosa Bouças, Jane H. Burridge, Margit Alt Murphy, Jaap Buurke, Peter Feys, Verena Klamroth-Marganska, Ilse Lamers, Gerdienke Prange-Lasonder, Annick Timmermans, Thierry Keller

**Affiliations:** 1 Faculty of Health Sciences, University of Southampton, Southampton, UK; 2 Department of Psychology, School of Health & Social Sciences, Buckinghamshire New University, High Wycombe, UK; 3 Institute of Neuroscience and Physiology, Rehabilitation Medicine, Sahlgrenska Academy, University of Gothenburg, Gothenburg, Sweden; 4 Roessingh Research and Development, Enschede, The Netherlands; 5 REVAL- Rehabilitation Research Center, BIOMED - Biomedical Research Institute, Faculty of Medicine and Life Sciences, Hasselt University, Hasselt, Belgium; 6 Sensory Motor Systems Lab, Department of Health Science and Technologies, Swiss Federal Institute of Technology, Zurich, Switzerland; 7 Department of Biomedical Signals and Systems, University of Twente, Enschede, The Netherlands; 8 Department of Mechanical Engineering, University of Twente, Enschede, The Netherlands; 9 Neurorehabilitation Department, Health Division, TECNALIA Research & Innovation, Donostia-San Sebastián, Spain

**Keywords:** Neurology, Assessment, Upper extremity, Rehabilitation technology, Robotics, Evaluation, Outcome measures

## Abstract

**Background:**

The need for cost-effective neurorehabilitation is driving investment into technologies for patient assessment and treatment. Translation of these technologies into clinical practice is limited by a paucity of evidence for cost-effectiveness. Methodological issues, including lack of agreement on assessment methods, limit the value of meta-analyses of trials. In this paper we report the consensus reached on assessment protocols and outcome measures for evaluation of the upper extremity in neurorehabilitation using technology. The outcomes of this research will be part of the development of European guidelines.

**Methods:**

A rigorous, systematic and comprehensive modified Delphi study incorporated questions and statements generation, design and piloting of consensus questionnaire and five consensus experts groups consisting of clinicians, clinical researchers, non-clinical researchers, and engineers, all with working experience of neurological assessments or technologies. For data analysis, two major groups were created: i) clinicians (e.g., practicing therapists and medical doctors) and ii) researchers (clinical and non-clinical researchers (e.g. movement scientists, technology developers and engineers).

**Results:**

Fifteen questions or statements were identified during an initial ideas generation round, following which the questionnaire was designed and piloted. Subsequently, questions and statements went through five consensus rounds over 20 months in four European countries. Two hundred eight participants: 60 clinicians (29 %), 35 clinical researchers (17 %), 77 non-clinical researchers (37 %) and 35 engineers (17 %) contributed. At each round questions and statements were added and others removed. Consensus (≥69 %) was obtained for 22 statements on i) the perceived importance of recommendations; ii) the purpose of measurement; iii) use of a minimum set of measures; iv) minimum number, timing and duration of assessments; v) use of technology-generated assessments and the restriction of clinical assessments to validated outcome measures except in certain circumstances for research.

**Conclusions:**

Consensus was reached by a large international multidisciplinary expert panel on measures and protocols for assessment of the upper limb in research and clinical practice. Our results will inform the development of best practice for upper extremity assessment using technologies, and the formulation of evidence-based guidelines for the evaluation of upper extremity neurorehabilitation.

**Electronic supplementary material:**

The online version of this article (doi:10.1186/s12984-016-0192-z) contains supplementary material, which is available to authorized users.

## Background

Assessment has been defined as a “detailed process which aims to define the nature and impact of an impairment and devise a treatment plan” [1]. Technologies are being developed for use in the assessment and treatment of patients with neurological conditions in both clinical and research environments [2, 3]. Development, funded by governments, research, and commercial organizations, is driven by the need for evidence-based neurological rehabilitation. But translation of new technologies into clinical practice is limited by a lack of evidence for effectiveness.

Methodological issues, including small sample sizes, lack of consensus on standardized assessment protocols and outcome measures, currently limit the value of meta-analyses of trials for rehabilitation of the upper extremity [4]. There is therefore an urgent need for agreed guidelines on measurement tools and assessment protocols. Furthermore, new technology-based measurement tools have the potential to be used alongside clinical measures of impairment, activity and participation, but need to be rigorously tested for usability, validity, reliability, and responsiveness. Agreement is needed on what parameters should be measured, using what tools (both clinical scales and technologies) and protocols for application; which assessments should be used in research and clinical practice, and when these assessments should be conducted.

The primary driver for this work was to improve effectiveness of upper extremity neurorehabilitation. Damage to the central nervous system such as stroke, multiple sclerosis (MS) or spinal cord injury (SCI) has an impact on arm function. It is estimated that only 41 % of people with moderate to severe stroke and 71 % with mild stroke regain dexterity [5] which is known to affect performance in activities of daily living (ADL) [6, 7]. Reduced hand dexterity and associated limitations in ADL as well as social activities have been identified as highly prevalent in mid and late stages of MS [8–10]. “Improving upper extremity recovery and function after stroke” [11], “identification of effective treatments to slow, stop or reverse the accumulation of disability associated with MS” [12] and “regaining arm/hand function after cervical SCI” [13] are main priorities identified by patients and carers. Wider effects are seen across society; in 2009 stroke alone was estimated to cost the EU economy over €38 billion with 50 % direct health care costs, 22 % productivity losses and 29 % to the informal care of people with stroke [14]. In 2005 the total annual cost of MS in Europe was estimated at €12.5 billion [15]. No European data was found for SCI, however in Australia, economic costs per patient were found to be higher for SCI than MS [16].

A positive relationship has been established between intensity and duration of therapy and outcomes [17]; a recent review suggested that strong evidence exists for physical therapy interventions favoring intensive highly repetitive task oriented and task-specific training in all phases post-stroke [18]. Governments, research and commercial organisations are investing in the development of rehabilitation technologies, cognisant that they are well placed to deliver this extra intensity, and have the potential to deliver cost-effective rehabilitation. However, translation of these technologies is limited by a lack of evidence for effectiveness and optimum delivery intensity, timing and duration. In addition to this, there is a need to identify which systems work best and for whom, which is only possible when clinical trial evidence with different systems and with patients having different impairment levels can be compared.

There are currently no standardised international evidence-based guidelines for either evaluation of upper extremity rehabilitation, or for technology-supported rehabilitation. Many published studies do not include adequate activity level or patient-reported outcome measures, which impede comparisons. A failure to measure these may have affected how the technologies were reported. Standardised assessment guidelines are needed to improve clinical practice, through better monitoring of patient progress and evaluation of treatment techniques. Agreed measures and protocols for assessment will enable data comparison across research trials, facilitating meta-analyses and lead to more robust evidence and consequently inform the design and development of new rehabilitation technologies.

Usefulness of consensus methods has been demonstrated in the development of clinical guidelines that define essential elements of the quality of healthcare [19–24]. Delphi methodology has been used to establish consensus in the absence of unanimity of opinion due to lack of scientific evidence or where the evidence is contradictory [25–35]. Features of the Delphi method include: anonymity (questionnaires are used to prevent dominant individuals exerting undue influence), iteration (processes occur in rounds to enable contributors to change their minds in response to the views of their peers), controlled feedback (showing the distribution of the group’s response), statistical group response (expressing judgment using summary measures of the full group response, giving more information than a single consensus statement) [25, 31, 35–37].

The traditional Delphi uses a series of sequential questionnaires with controlled feedback [37]. A modified Delphi consensus method has been applied in a variety of ways [23, 24, 38] e.g., using an iterative process with qualitative open-ended questioning in all rounds [39] or using a checklist to which participants respond instead of a first round questionnaire [40]. There is no empirical evidence to guide the identification of the specific content of evidence-based guidelines for assessment. In such cases consensus studies with experts have been advocated as the “next best” option [34].

### Aim

The aim of this research was to achieve European-wide consensus on the evaluation of the upper extremity in neurorehabilitation using technology. The consensus will recommend a framework for assessment, including, where possible, specific measures and how and when they should be used in clinical practice and research. The paper describes the modified Delphi methodology and presents the outcome of this rigorous iterative process through which consensus was reached among a panel of international multidisciplinary experts. The outcomes of this research will be combined with other data sources and used to create European guidelines for clinicians and researchers.

## Methods

### Recruitment

Monitoring and advisory groups were initially formed. The purpose of the monitoring group was to oversee the Delphi technique, to define the rules of engagement, the process of data collection, and the criteria for consensus (these will be explained in more detail in subsequent sections). Monitoring members were either experts in using rehabilitation technologies for assessment and treatment and members of the European Cooperation in Science and Technology (EU COST) Action TD1006 (2011–2015) European Network on Robotics for NeuroRehabilitation, or experts in the Delphi methodology. The purpose of the advisory group was to participate in the ideas generation rounds, and to contribute in the design and piloting of the questionnaire. Advisory members were professionals with expertise in using technologies for assessment and management of neurological conditions and members of the above mentioned EU COST Action TD1006.

Meeting convenors (volunteers from the EU COST Action TD1006 membership) contacted their professional networks and invited those interested who met the inclusion criteria to join the consensus expert groups. The inclusion criteria were: self-reported experience in neurological assessments or technologies, employed in European institutions, and different professional backgrounds to allow the creation of two major groups: i) practicing clinicians who treat patients as the focus of their daily work (e.g., therapists, medical doctors, etc.) and ii) researchers (clinical researchers, non-clinical researchers e.g. movement scientists, technology developers and engineers). The experts were all self-selected based on their interest in the subject area.

### Delphi procedure

A Modified Delphi consensus exercise was implemented in three stages. The monitoring group decided that fewer rounds were necessary for Stages 1 and 2 as these were preparation stages for the consensus exercise in Stage 3.

Stage 1- Ideas generation (three rounds): This aimed to identify principal factors with regard to assessment, for example, defining the purpose of assessment, the sort of and timing of technological and traditional outcome measures. The domains considered included impairments at the body and body part level, person level activity limitations, and societal level restrictions of participation [41].

Stage 2 - Design and piloting of consensus questionnaire (two rounds): This aimed to: generate further questions based on the minutes of previous meetings; describe the Delphi methodology to the team and to pilot initial questions/statements. The following rules were adopted to build the questionnaire. Multiple choice questions/statements were used to try and identify which specific measures should be used. A statement would include what would be measured; the choices would include the specific measures to choose from. Participants would then choose the measures they deemed appropriate to measure the specific construct. When there was lack of consensus following discussion questions were reworded for clarification or changed to generic “Yes or No” questions based on the discussion that had taken place. Piloting was also used to refine the definition of consensus prior to the consensus rounds [25, 42, 43] and to ensure rigour in the design of first-round questions [44] and the choice of measurement methods and their analysis in subsequent rounds [25, 45].

Stage 3- Consensus (five rounds): This aimed to identify which statements consensus could be gained on. To do this, background information on the guidelines, Delphi methodology and the rules of engagement were explained to the participants; specifically, that the Delphi method was chosen in order to reach a consensus on outcome measure recommendations using a 3-stage data collection process. Participation was irrespective of whether the experts had taken part in the previous round.

### Data collection and voting

The feasibility of using an anonymous audience response system (ARS; TurningPoint Technologies, Youngstown, OH, USA) to enable polling using a PowerPoint 2010 presentation and electronic voting devices (zappers) was established in stage 2. Voting was undertaken in stage 3. Where no consensus was reached there was a subsequent discussion and a second round of voting which either achieved consensus, or generated new questions, or informed amendments to existing questions to facilitate the gaining of consensus in the next round. Members of the monitoring committee were responsible for instigating discussions on a statement by statement basis and recording comments expressed by the experts during those discussions using a tablet. There was a maximum time limit of 15 min on the discussion time of any individual statement. The moderator was the Project Investigator of the EU Cost Action Group.

### Consensus procedure

The reported level of agreement which constitutes consensus varies [25, 34], but is generally recommended to be set at an agreed threshold of 70 % or above prior to commencing the study, with the potential to change with subsequent rounds [25, 46]. A threshold of 75 % or higher of participants voting on a particular answer was set for Round 1. Agreement reaching the threshold would result in the statements being excluded from subsequent rounds and included in the guidelines. Where agreement on statements did not reach this threshold, the statements would be amended as mentioned above. This procedure of re-evaluation continued until either the consensus rate was achieved or until the Delphi panel members no longer modified their previous responses (or comments). In those cases when both the level of agreement and the type of comments on the re-entered questions no longer changed it was agreed that a further round would not achieve consensus. The comments and suggested additions were collated and reviewed for consistency and overlap by the monitoring group. Inconsistent or overlapping additions were omitted; the others were developed for consideration in the following consensus round.

In analysing the data, and in understanding the difficulty of reaching consensus in the latter rounds where iteration had featured, a pragmatic decision was taken by the monitoring group to lower the threshold marginally to 69 % (total participant response). This met published criteria that consensus is achieved when 66.6 % of a Delphi panel agrees [47].

### Analysis of responses

Two groups comprising clinicians and researchers (clinical and non-clinical researchers e.g. movement scientists, technology developers, and engineers) were considered for analysis, to inform the two proposed guidelines – one for clinicians and one for researchers. The percentage of participants who voted for each possible answer was calculated for all questions in all five rounds.

## Results

The monitoring group consisted of two clinical researchers (JHB, AMH) and one Delphi expert (SBB). The advisory group consisted of 13 professionals: three clinicians and ten researchers (six clinical researchers, three non-clinical researchers, one engineer). The composition of the expert groups is shown in Table 1.

**Table 1:** Composition of monitoring, advisory and consensus expert groups

Location	Date	Expert group total	Practising clinicians^a^	Researchers		
				Clinical researchers^b^	Non-clinical researchers^c^	Engineers^d^
Ideas generation rounds						
Brussels	25/11/2011	13	3	6	3	1
Southampton	19/03/2012	41	3	12	0	26
Brussels	28/04/2012	9	1	5	1	2
Design and piloting of the questionnaire meetings						
Brussels	07/11/2012	8	1	6	0	1
Brussels	08/11/2012	12	3	6	3	0
Delphi rounds						
Madrid(Round 1)	13/11/2012	34	3	3	26	2
Bucharest (Round 2)	26/03/2013	35	26	3	6	0
Enschede (Round 3)	08/04/2013	71	12	14	27	18
Pisa (Round 4)	10/05/2013	43	15	11	11	6
San Sebastian (Round 5)	05/06/2013	25	4	5	7	9
Total for Delphi rounds		208	60	35	77	35

^a^Practising clinicians defined as those who treat patients as the focus of their daily work (e.g., therapists, medical doctors, etc.)

^b^Clinical researchers defined as clinicians whose focus is on research

^c^Non-clinical researchers defined as researchers with no healthcare qualification (e.g. movement scientists)

^d^Engineers defined as technology developers or engineers

### Delphi procedure outcomes

#### Stage 1 - Ideas generation

Three preliminary meetings took place: the first with 13 contributors from 8 European countries, the second with 41 EU COST Action Group members from 22 countries, and the third with nine contributors from six countries (Table 1). Initial discussions focused on potential usefulness of guidelines, and identification of a suitable methodology to establish them. The definition and purpose of assessment, outcome measures currently used and those that have the potential to be used were discussed (Table 2) and consolidated into a format suitable for a questionnaire.

**Table 2:** Ideas Generation Rounds – Discussion topics

Usefulness of guidelines in technology-based neuro-rehabilitation	
What is the aim in generating and publishing the guidelines and how these will be used?	
What is the purpose of measurement?	
Should recommendations be based on the ICF Framework and include measures in each category?	
Should preference be given to measures that span more than one ICF category?	
Are the following measures are useful for clinicians and researchers? If so, which measures/information/variables?	
Technology-generated data EMG, cortical or eye movement, measures of impairment, activity, participation measures	

#### Stage 2 – Design and piloting of the consensus questionnaire

**Fig. 1:**
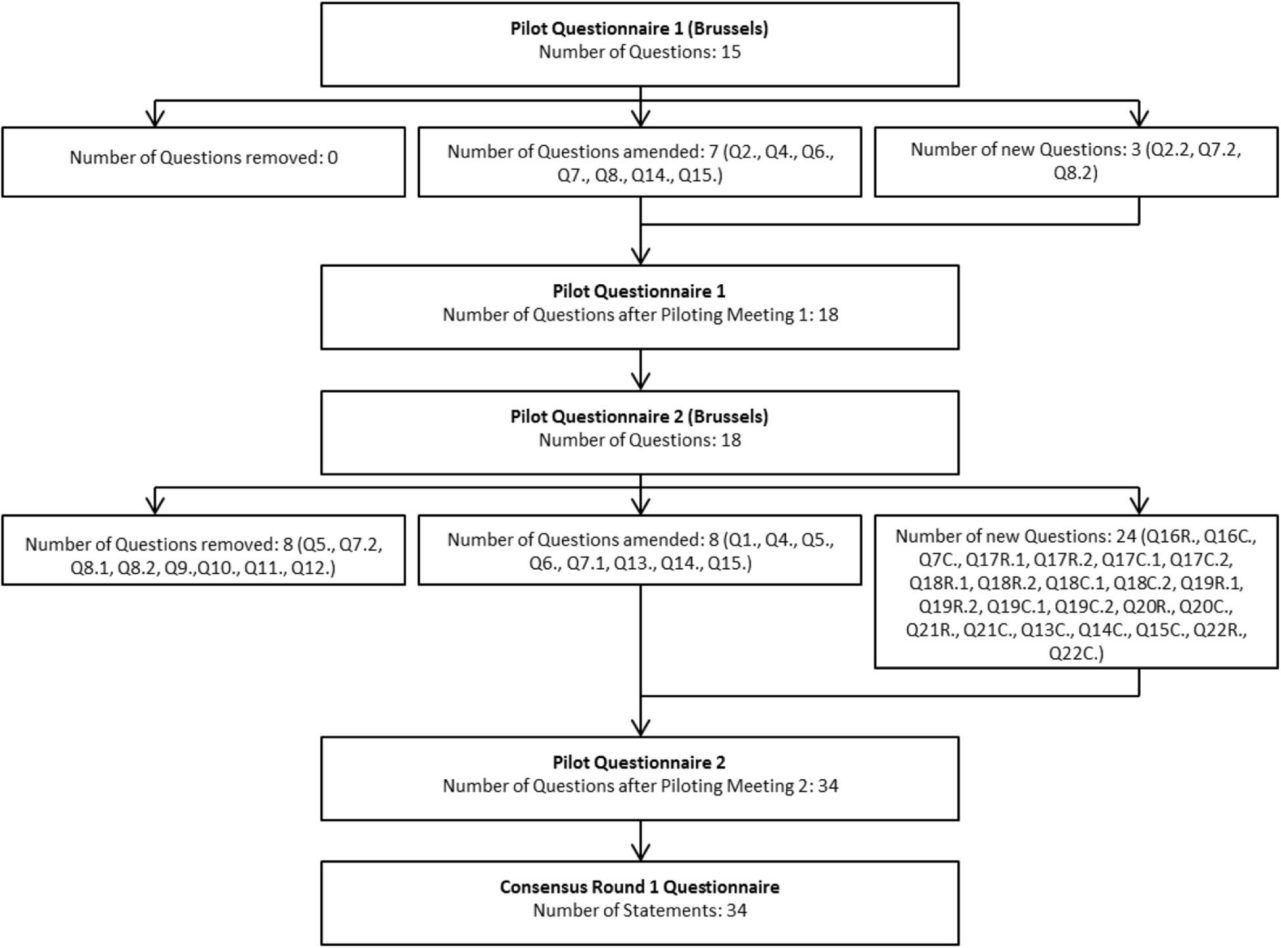
Flowchart of the design and piloting of the questionnaire

The feasibility of using the TurningPoint software and electronic voting devices (zappers) as a method of achieving consensus was established by the monitoring and advisory group piloting initial statements/questions (*n* = 15). Issues with the format of several questions including the possibility for response bias, multiple questions, or lack of specificity were identified and the questionnaire was revised. This was again piloted, discussed and refined. On the basis of discussion, new topics were included, questions were reformatted to allow for separate guidelines for research and clinical areas. This process developed the statements (*n* = 34) for Round 1 of the consensus exercise. The piloting flow chart (Fig. 1) shows how many questions were removed, amended to facilitate understanding or added at each stage.

C&R indicate whether the question/statement is applicable to practice in clinic (C) or to research (R).

#### Stage 3 – Consensus

**Fig. 2:**
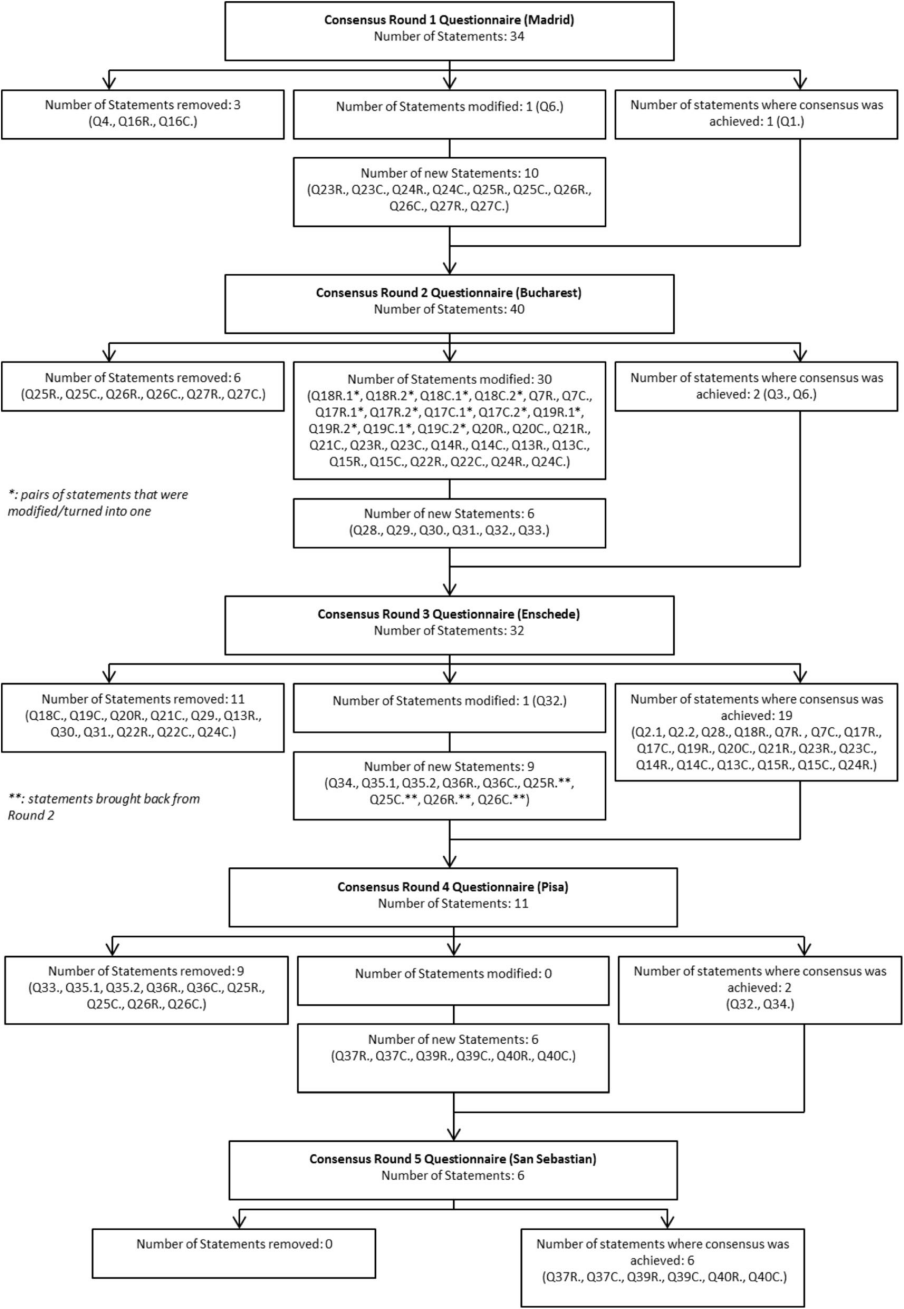
Flowchart of the Consensus Rounds

For the statements in which no consensus was achieved, on the basis of discussion, new topics were included, and questions were reformatted. The consensus flow chart (Fig. 2) shows how many questions were removed, amended to facilitate understanding or added at each stage.

C&R indicate whether the question/statement is applicable to practice in clinic (C) or to research (R).

In total 65 statements were considered. For ease of viewing, the statements relating to clinicians and researchers, have been combined resulting in 22 consensus statements (Table 3) and 20 statements where consensus was not achieved (Table 4).

**Table 3:** Consensus statements

Round	Question number	Statements	Agreement overall % (n)	Agreement clinicians % (n)	Agreement researchers % (n)
1	Q1.	Recommendations on an assessment framework and outcome measures for use in technology based neuro-rehabilitation are useful.	79 (27)	100 (3)	77 (24)
2	Q3.	The purpose of measurement is to design therapy (initial decisions and changes in therapy programme) and to measure progress.	86 (30)	81 (21)	100 (9)
2	Q6.	Technology-generated data (from a device, wearable and environmental sensors, wii, social networking, etc.) should be used by both clinicians and researchers.	83 (29)	81 (21)	89 (8)
3	Q2.1. Q2.2	Recommendations including a minimum defined set of measures should be used by:			
		Clinicians	83 (59)	100 (12)	80 (47)
		Researchers	87 (62)	100 (12)	85 (50)
3	Q28.	Measures that are not currently widely used or practical, but which have the potential to be useful should be included.	92 (65)	92 (11)	92 (54)
3	Q18R.	Performance quality measures (Co-ordination, smoothness of movement, precision/accuracy of movement, number of errors and successes - during the performance of a task), Response to perturbations (disturbances during movement), and Compensatory (abnormal) movements should be used by researchers.	73 (55)	64 (7)^a^	75 (48)
3	Q7C. Q7R.	A range of kinematic measures (active ROM at a single joint, extent of workspace at multiple joints, speed of movement, etc.) should be used by:			
		Clinicians	76 (54)	75 (9)	76 (45)
		Researchers.	83 (59)	75 (9)	85 (50)
3	Q17C. Q17R.	A range of kinetic measures (e.g., Isometric force in a range of muscles and positions, Isokinetic force in a range of muscles and movements, endurance, Grip strength, Non-neural muscle stiffness (resistance to passive movement), and Spasticity) should be used by:			
		Clinicians	53 (40)^a^	79 (11)	47 (29)^a^
		Researchers.	55 (56)^a^	77 (10)	52 (46)^a^
3	Q19R.	A range of EMG measures (e.g., Co-contraction, Synergies, Muscle onset time, and Inappropriate muscle activity, etc.) should be used by researchers.	83 (59)	91 (10)	82 (49)
3	Q20C.	Effort during movement measure (amount of assistance required to complete task) should be used by clinicians.	73 (48)	67 (8) ^a^	74 (40)
3	Q21R.	A range of neuropsychological and other non-motor domain measures (e.g., Attention +/− when distracted, Neglect, Engagement, Reaction time, and Pain associated with movement, etc.) should be used by researchers.	100	N/A^b^	100 (59)
3	Q23C. Q23R.	Non-technology based measures of Impairment should be restricted to validated outcome measures (e.g. Fugl Meyer (FM), Grip or muscle strength), except in certain circumstances (e.g., experimental research, development of new technologies or for the purposes of validation) for use of:			
		Clinicians	76 (54)	75 (9)	76 (45)
		Researchers	71 (44)	80 (8)	69 (36)
3	Q14C. Q14R.	Technology-based measures of Activity (e.g., Measures that can be used at home, Body-worn sensors to monitor activity, and Body-worn sensors as surrogate measures of function (e.g., the WMFT)) should be used by:			
		Clinicians	53 (31)^a^	50 (6)^a^	54 (25)^a^
		Researchers	72 (39)	70 (7)	73 (32)
3	Q13C.	Non-technology based measures of Activity should be restricted to validated outcome measures such as the Action Research Arm Test (ARAT) or Wolf Motor Function Test (WMFT) for use of clinicians.	72 (51)	67 (8) ^a^	73 (43)
3	Q15R.	In research, non-technology based Measures of Participation measures should be restricted to validated outcome measures such as the Stroke Impact Scale (SIS), Short Form-36 (SF36), except in certain circumstances (e.g., experimental research, development of new technologies or for the purposes of validation).	85 (45)	71 (5)	87 (40)
3	Q15C.	In clinic, non-technology based Measures of Participation measures should be restricted to validated outcome measures such as the Stroke Impact Scale (SIS), Short Form-36 (SF36).	70 (50)	75 (7)	70 (41)
3	Q24R.	A range of Neurophysiology Measures (e.g., EEG, TMS/MEP, and functional neuro-imaging) should be used by researchers.	76 (47)	100 (9)	72 (38)
4	Q32.	Self-reported measures (e.g. Motor Activity Log) should be used.	91 (39)	93 (14)	89 (25)
4	Q34.	Personalized, goal orientated measures (e.g. COPM) should be used.	84 (37)	81 (13)	86 (24)
5	Q37R. Q37C.	Four face-to-face patient assessments should be collected for a treatment programme: Baseline (beginning of the programme), interim (during the programme), final (end of the programme), and follow-up (a set period of time after the end of the programme), other than data collected automatically by technology for:			
		Clinicians	100 (24)	75 (3)	100 (21)
		Researchers	83 (20)	75 (3)	81 (17)
5	Q39R. Q39C.	Assessment should take place separately from treatment by:			
		Clinicians	96 (23)	100 (4)	90 (19)
		Researchers.	83 (20)	75 (3)	81 (17)
5	Q40R. Q40C.	Maximum time for assessment is 3 h for:			
		Clinic	92 (22)	75 (3)	90 (19)
		Research	83 (20)	75 (3)	81 (17)

C&R indicate whether the question/statement is applicable to practice in clinic (C) or to research (R)

^a^: The monitoring group agreed that the statement should be accepted as having reached consensus for the following reasons: These was clear agreement from practicing clinicians, and the majority of non-clinicians and non-practicing clinicians also voted for this option or the majority of experts voted for this option (the remaining votes were spread in support of the different individual measures which are encompassed by this statement)

^b^: Practicing clinicians did not vote

**Table 4:** Statements for which consensus was not achieved

Round	Question number	Statements	Average agreement for all options overall %
1	Q4.	Recommendations (including technology-based and clinical measures) should fall within the ICF Framework.	25
1	Q16R. Q16C.	Which categories – kinematic, kinetic, quality of movement, effort, and neuropsychological and other non-motor domain measures are most important for researchers and clinicians.	20 (R) 20 (C)
2	Q25R. Q25C.	The amount of time researchers and clinicians would be willing to spend on using outcome measures for assessment (beginning and end of seeing patient).	14 (R) 14 (C)
2	Q26R. Q26C.	The frequency researchers and clinicians would be willing to evaluate the patient’s progress.	17 (R) 17 (C)
2	Q27R. Q27C.	The amount of time researchers and clinicians would be willing to spend on using outcome measures for evaluation of patient progress.	14 (R) 14 (C)
3	Q18C.	Which Quality of Movement Measures should be recommended for clinicians to use.	20
3	Q19C.	Which Muscle Activity Measures (EMG) should be recommended for clinicians to use.	17
3	Q20R.	Which Effort During Movement Measures should be recommended for researchers to use.	20
3	Q21C.	Which Neuropsychological and other non-motor domain measures should be recommended for clinicians to use.	14
3	Q29.	Which non-technology-based Measures of Impairment should be recommended.	20
3	Q13R.	In research, non-technology-based Measures of Activity should be restricted to validated outcome measures such as the Action Research Arm Test (ARAT) or Wolf Motor Function Test (WMFT).	20
3	Q30.	Which non-technology-based Measures of Activity should be recommended.	17
3	Q31.	Which non-technology-based Measures of Participation should be recommended.	25
3	Q22R. Q22C.	Which categories of technology-based Measures of Participation should be recommended for researchers and clinicians to use.	20 (R) 20 (C)
3	Q24C.	Which Neurophysiology Measures should be recommended for clinicians to use.	20
4	Q33.	Which Self-Reported Measures should be recommended.	33
4	Q36R. Q36C.	The minimum frequency of face-to-face patient assessment (other than data collected automatically by technology) for researchers and clinicians.	25 (R) 25 (C)
4	Q25R. Q25C.	The amount of time researchers and clinicians are to spend in face-to-face assessment at the beginning and end of a treatment programme.	20 (R) 20 (C)
4	Q26R. Q26C.	The amount of time researchers and clinicians are to spend in spend in face-to-face assessment of patients’ progress.	20 (R) 20 (C)

C&R indicate whether the question/statement is applicable to practice in clinic (C) or to research (R)

### Consensus statements outcomes

Key areas of consensus were established for both clinicians and researchers (Table 3). The expert population surveyed agreed that the publication of recommendations on an assessment framework and outcome measures for use in technology based neurorehabilitation would be useful. An agreed definition of the purpose of measurement was established. Clinicians and researchers agreed that a minimum defined set of measures (both currently available and those with future potential) should be used.

Agreement was reached on standardising patient assessments to a minimum of four face-to-face assessments for a treatment programme: baseline (beginning of the programme), interim (during the programme), final (end of the programme), and follow-up (a set period of time after the end of the programme), which should take place separately from treatment and last no longer than three hours was thought to be clinically important as well as achievable.

The expert population agreed that technology-generated data (e.g. kinematic, kinetic and activity measures) should be used whilst non-technology based measures should be restricted to validated outcome measures except in certain circumstances for research (for example if they were validating a new outcome measure). Other measures which were recommended to be included were self-report and personalized goal-oriented measures.

Specific agreement was reached for measures to be used by researchers including quality of movement, EMG, neurophysiological measures and neuropsychological and other non-motor domain measures including attention, neglect, engagement, reaction times and pain). Specific agreement reached for measures to be used by clinicians included patient effort and non-technology measures of activity.

There was agreement to include existing clinical outcome measures in clinical practice (e.g. the Action Research Arm Test) but experts did not agree on the need to limit research studies to these outcome measures only. Statements that were excluded (Table 4) were frequently those in which specific outcome measures or times for assessment were suggested.

## Discussion

Technologies can provide valid, reliable and sensitive assessment tools that, when used alongside clinical measures, can inform clinical decision-making and provide richer data on patient outcomes. There is now a clear need for guidelines for clinicians and researchers to optimise technology-based assessment and application of clinical measures and procedures. This paper reported the consensus of a panel of experts and the process through which it was reached. It will inform clinical and research evidence-based guidelines for evaluation of technology-based upper extremity neurological rehabilitation.

Using the modified Delphi technique, we gained consensus from 208 European participants across multi-disciplinary professional expert groups including both practicing clinicians and researchers. In general, clinicians and researchers agreed that: i) recommendations on assessments for use in technology-based neurorehabilitation would be useful; ii) the purpose of measurement is to design therapy and to measure progress; iii) a minimum defined set of measures should be used; iv) the minimum number of, timing and duration of assessments should be defined; v) technology-generated assessments should be used by both clinicians and researchers in conjunction with clinical assessments which should be restricted to validated outcome measures (except in certain circumstances for research). Self-reported and personalized goal-oriented measures were also recommended to be added in the guidelines.

Excluded statements were frequently those in which specific outcome measures or times for assessment were suggested. In discussion surrounding these points, the consensus expert groups suggested this may be a reflection on the practicalities of what can be achieved given the resource issues affecting most health services. Also, whilst it might have been expected that clinical assessments should take less time than quantitative research assessments, the discussion included the issue that nerve conduction tests might be performed within clinical assessments. This work has delivered the largest “expert consensus” view within the field with good multidisciplinary representation, which we consider will be critical to future adoption of the guidelines by clinicians and researchers.

The modified Delphi technique has recently been used successfully in the development of a tool to assess quality of stroke care across European populations [48], to identify a set of clinically useful outcome measures for assessment of adults receiving treatment for spasticity [49] and to develop a post-stroke checklist to standardize follow-up care for stroke survivors [50]. The modified Delphi technique used in the current research was found to be a flexible and adaptable tool to gather and analyse relevant data from the European cross-disciplinary groups.

The statements with the highest overall agreement were on the usefulness of the guidelines, recommendations about the duration and timing of assessments and the recognition that given the speed of technology change, the guidelines should be written to allow inclusion of future potentially useful measures. The need for assessments is emphasised within many healthcare professional training programmes, however there is scant detail on how to choose and implement such assessments in international clinical guidelines, which is perhaps why this was viewed as such an important subject on which to reach consensus.

Agreement was reached on standardising patient assessments to a minimum of four face-to-face assessments for a treatment programme at: baseline (beginning of the programme), interim (during the programme), final (end of the programme), and follow-up (a set period of time after the end of the programme). It was agreed that these assessments should take place separately from treatment and last no longer than three hours which was thought to be clinically important as well as achievable. It is recognised that variation exists in what is supported in clinical practice in European healthcare systems. In some countries, costs for the inclusion of an assessment phase during clinical practice follow-up are not supported. In others, even though active rehabilitation has ended, people still have check-ups with their rehabilitation physician for monitoring purposes, at least for the first year after stroke. In clinical practice, practical issues (such as transfers of patients to home, other wards or hospitals) may reduce the number of post baseline assessments from occurring. However, these assessments are essential for tailoring treatments and increasingly to financially justify therapy by providing evidence for the cost benefit of a rehabilitation programme. With increasingly stretched resources there is likely to be greater emphasis on being able to demonstrate value for money in the future.

The only formalised practice guidelines on stroke rehabilitation to explicitly address specific assessments, within the authors’ knowledge, are the Dutch Stroke Guidelines which are issued by the Royal Dutch Society of Physical Therapy [51]. The Dutch Stroke Guidelines state a minimal and supplemental set of clinical outcome measures along with recommendations for when these should be recorded (in the week of admission and of discharge, before multidisciplinary meetings and at the end of the 1st week, 3rd month and 6th month post-stroke). The Dutch Guidelines also state that if patients are continuing treatment during the chronic phase, monthly evaluations are advised. Adoption of the guidelines should ensure that whatever the practical issues, the same assessments measures are used.

Many meta-analyses and systematic reviews of research trials have commented on the lack of consistency of outcome measures, and highlighted that it would be useful if outcome measures of arm function and measures of repetitions during training could be used in future studies to gain a better understanding of the effects of training [4, 52]. Within this Delphi study, consensus was lacking for statements in which we tried to select specific measures to recommend. This may reflect either the impracticality of using specific measures, a desire to not be restricted to using specific measures, differing education or practices throughout Europe, a lack of awareness of the current research evidence or just different opinions. The work of this study is complemented by a recent Italian national Delphi consensus on specific outcome measures to be used specifically for evaluating robot assisted rehabilitation after stroke. It identified eight clinical scales for evaluation of the upper limb and ten clinical scales for evaluation of the lower limb [53]. The paper indicates that differing educational and/or practicing cultures among multiple countries may be an important issue. Additionally, this may point to difficulty experienced among experts to specify a restricted set of outcome measures for a rather broad field of application, covering the full range of neurorehabilitation (addressing multiple disorders, involving a variety of conventional techniques as well as emerging technology-assisted methods).

The high consensus for the inclusion of measures not currently widely used or practical, but which have the potential to be useful, pending technological innovation, reflects a recognition by researchers and clinicians that current assessment tools are inadequate and that there is a need for change. Technology can generate high quantities of data. It is difficult to know what data will provide therapists with the most useful information for treatment planning for patients. Movement duration and smoothness for example have been found to be associated with real clinical improvement in upper limb function [54]. Technology-based measures now need to be incorporated into easy to use clinical and home-based rehabilitation systems to facilitate the continuity of objective assessments allowing better longer term self-management. This study provides a mandate for this.

### Strengths and limitations

A multidisciplinary panel of clinicians, non-practicing clinicians and non-clinicians from over 23 countries have contributed to consensus on assessments and assessment protocols. The rigorous modified Delphi technique enabled questions and statement to be honed and simplified and potential misinterpretations to be identified and revised. The initial aim, as previously stated, was to achieve a consensus recommending a framework for assessment, including, where possible, specific measures and how and when they should be used in clinical practice and research. To try and achieve this, initially questions and statements were very detailed and were in some cases ambiguous, which led to a lack of consensus. The process ensured that these questions and statements were adapted to become unambiguous and more generic, providing practical guidance without compromising professional autonomy.

A comparable Delphi survey [55] reported potential linguistic misinterpretation of questions and statements by a multi-lingual panel as a limitation. We mitigated for this potential risk by using an advisory group representing each profession and comprising eight nationalities and six different first languages, but all fluent English speakers, to develop and agree upon the initial statements and questions. Throughout the consensus process we were also careful to explain each question and statement and ask participants if they understood before proceeding. However, given the multidisciplinary, multi-lingual membership of the expert groups, the potential for misinterpretation remained. It is also noted that use of a different form of data collection (e.g. a written questionnaire administered by e-mail) may have produced different results.

### Impact on future research and clinical practice

Our results will be combined with information on current published guidelines and a systematic review of the literature [56], to form European evidence-based clinical and research guidelines for the evaluation of technology-based upper extremity neurorehabilitation.

The guidelines, an output from the EU COST Action TD1006 (2011–2015) European Network on Robotics for NeuroRehabilitation, will have an impact on upper limb neurorehabilitation research by promoting well-informed and agreed standards for selection of measurement tools and protocols for assessment. If adopted they will underpin comprehensive data comparison across research trials, facilitating meta-analyses which will consequently improve evidence. The results of this consensus study will also inform clinical practice, allowing for improved assessments, better-informed clinical decision making, and thus choice of intervention and systematic monitoring of patient progress and evaluation of individual treatment techniques and potentially better patient outcomes (Additional file 1).

## Conclusion

The modified Delphi technique was found to be a flexible and adaptable tool to gather and analyse data from a large international multidisciplinary expert panel on measures and protocols for assessment of the upper limb in research and clinical practice. The main consensus points included:

- Recognition of the need for guidelines on the evaluation of the upper extremity neurorehabilitation using technology
- Standardising patient assessments to a minimum of four face-to-face assessments for a treatment programme: baseline (beginning of the programme), interim (during the programme), final (end of the programme), and follow-up (a set period of time after the end of the programme). These assessments should take place separately from treatment and last no longer than three hours
- Clinical assessments should be restricted to validated outcome measures
- Technology-generated assessments should be used in conjunction with clinical assessments
- Self-reported and personalized goal-oriented measures should also be included
- Measures which have the potential to be useful in the future due to technological progression should be included. Researchers and clinicians recognise that current assessment tools are inadequate to assess in detail the full spectrum of upper limb function, and that there is a need for change as new technologies become more widely available.

Addressing these will positively impact both research and clinical practice. Our results will inform the development of best practice for upper extremity assessment using technologies, and the formulation of evidence-based guidelines for the evaluation of upper extremity neurorehabilitation.

## Additional file

Additional file 1: Data from Delphi rounds. (XLSX 246 kb)

## Abbreviations

ADL: Activities of daily living
EU COST: European Cooperation in Science and Technology
MS: Multiple sclerosis
SCI: Spinal cord injury
